# Cumulative exposure to environmental pollutants during early pregnancy and reduced fetal growth: the Project Viva cohort

**DOI:** 10.1186/s12940-018-0363-4

**Published:** 2018-02-20

**Authors:** Lisa B. Rokoff, Sheryl L. Rifas-Shiman, Brent A. Coull, Andres Cardenas, Antonia M. Calafat, Xiaoyun Ye, Alexandros Gryparis, Joel Schwartz, Sharon K. Sagiv, Diane R. Gold, Emily Oken, Abby F. Fleisch

**Affiliations:** 1 Division of Chronic Disease Research Across the Lifecourse, Department of Population Medicine, Harvard Medical School and Harvard Pilgrim Health Care Institute, 401 Park Drive, Suite 401, Boston, MA 02215 USA; 2 Department of Biostatistics, Harvard T.H. Chan School of Public Health, Boston, MA USA; 3 Division of Laboratory Sciences, National Center for Environmental Health, Centers for Disease Control and Prevention, Atlanta, GA USA; 4 Department of Hygiene, Epidemiology and Medical Statistics, University of Athens Medical School, Athens, Greece; 5 Department of Environmental Health, Harvard T.H. Chan School of Public Health, Boston, MA USA; 6 Center for Environmental Research and Children’s Health, University of California, Berkeley, CA USA; 7 Division of Epidemiology, University of California, Berkeley School of Public Health, Berkeley, CA USA; 8 Channing Division of Network Medicine, Brigham and Women’s Hospital and Harvard Medical School, Boston, MA USA; 9 Department of Nutrition, Harvard T.H. Chan School of Public Health, Boston, MA USA; 10 Pediatric Endocrinology and Diabetes, Maine Medical Center, Portland, ME USA; 11 Center for Outcomes Research and Evaluation, Maine Medical Center Research Institute, Portland, ME USA

**Keywords:** Pregnancy, Birth weight, Epidemiology, Air pollution, Smoking, Perfluoroalkyl substances

## Abstract

**Background:**

Reduced fetal growth is associated with perinatal and later morbidity. Prenatal exposure to environmental pollutants is linked to reduced fetal growth at birth, but the impact of concomitant exposure to multiple pollutants is unclear. The purpose of this study was to examine interactions between early pregnancy exposure to cigarette smoke, traffic pollution, and select perfluoroalkyl substances (PFASs) on birth weight-for-gestational age (BW/GA).

**Methods:**

Among 1597 Project Viva mother-infant pairs, we assessed maternal cigarette smoking by questionnaire, traffic pollution at residential address by black carbon land use regression model, and plasma concentration of select PFASs in early pregnancy. We calculated sex-specific BW/GA z-scores, an index of fetal growth, from national reference data. We fit covariate-adjusted multi-pollutant linear regression models and examined interactions between exposures, using a likelihood-ratio test to identify a best-fit model.

**Results:**

Two hundred six (13%) mothers smoked during pregnancy. Mean [standard deviation (SD)] for black carbon was 0.8 (0.3) μg/m^3^, perfluorooctane sulfonate (PFOS) was 29.1 (16.5) ng/mL, and BW/GA z-score was 0.19 (0.96). In the best-fit model, BW/GA z-score was lower in infants of mothers exposed to greater black carbon [− 0.08 (95% CI: -0.15, − 0.01) per interquartile range (IQR)]. BW/GA z-score (95% CI) was also lower in infants of mothers who smoked [− 0.09 (− 0.23, 0.06)] or were exposed to greater PFOS [− 0.03 (− 0.07, 0.02) per IQR], although confidence intervals crossed the null. There were no interactions between exposures. In secondary analyses, instead of PFOS, we examined perfluorononanoate (PFNA) [mean (SD): 0.7 (0.4) ng/mL], a PFAS more closely linked to lower BW/GA in our cohort. The best-fit multi-pollutant model included positive two-way interactions between PFNA and both black carbon and smoking (p-interactions = 0.03).

**Conclusions:**

Concurrent prenatal exposures to maternal smoking, black carbon, and PFOS are additively associated with lower fetal growth, whereas PFNA may attenuate associations of smoking and black carbon with lower fetal growth. It is important to examine interactions between multiple exposures in relation to health outcomes, as effects may not always be additive and may shed light on biological pathways.

**Electronic supplementary material:**

The online version of this article (10.1186/s12940-018-0363-4) contains supplementary material, which is available to authorized users.

## Background

Fetal growth restriction is a major public health challenge, as it occurs in 5 to 10% of pregnancies and is one of the leading causes of neonatal mortality and morbidity [1]. Smaller weight for gestational age at birth, an indicator of fetal growth restriction, not only influences early-life outcomes, but is associated with adverse cardiometabolic health later in life [2].

Several in utero environmental exposures, many of which are modifiable, are linked to reduced fetal growth. For example, prenatal exposures to cigarette smoke, traffic pollution, and perfluoroalkyl substances (PFASs) have been associated with lower fetal growth in our greater Boston area longitudinal birth cohort, Project Viva [3–5], as well as in other epidemiological studies [6–10]. Cigarette smoke and traffic-related air pollution contain particulate matter (PM), along with hydrocarbons, aldehydes, nitric oxides, and carbon monoxide [11]. Both exposures are hypothesized to lower fetal growth primarily as a result of placental oxidative stress and inflammation [12, 13], which may disturb nutrient and oxygen exchange from mother to fetus [12]. PFASs are synthetic compounds used in the manufacture of a variety of consumer and industrial products, including water- and stain-resistant coatings for clothing, furniture, and carpets; oil-resistant coatings for food packaging; non-stick cookware; fire-fighting foams; and aviation hydraulic fluids. Almost the entire U.S. population (> 95%) has detectable serum concentrations of several PFAS [14–16]. PFASs may influence fetal growth by activating the peroxisome proliferator-activated receptor (PPAR) [17], which may inhibit inflammatory gene expression [18, 19] and interfere with lipid and glucose homeostasis [19, 20].

Although studies have reported robust associations of each of these prenatal exposures with reduced fetal growth, few prior studies have evaluated the potential impact of these pollutants combined. This is important because pregnant women are simultaneously exposed to mixtures of environmental toxicants [21].

In the present analysis, we examine additive interactions between early pregnancy exposure to cigarette smoke, black carbon (an indicator of traffic pollution), and select PFASs and their associations with birth weight-for-gestational age (BW/GA) in the Project Viva cohort. Based on our prior work in subsets of this cohort, we expected to find that each exposure would be independently associated with fetal growth and further hypothesized that infants exposed to all three would have the lowest expected fetal growth.

## Methods

### Study population and design

Between 1999 and 2002, we recruited pregnant women into the Project Viva cohort during their initial prenatal visit (median 9.9 weeks gestation) at Atrius Harvard Vanguard Medical Associates, a multi-specialty group practice with offices throughout eastern Massachusetts. Exclusion criteria included multiple gestation, inability to answer questions in English, gestational age greater than or equal to 22 weeks at recruitment, and plans to move from the area prior to delivery [22]. A total of 2128 women were initially included in the cohort. In the present analysis, we excluded one participant without a recorded birth weight, 21 without information on prenatal cigarette smoking, 37 without data on estimated residential black carbon concentration, one for whom the black carbon regression model provided an implausible data point, and 471 whose mother did not provide a plasma sample or did not have sufficient sample for quantification of PFASs and thus did not have a measurement of maternal perfluorooctane sulfonate (PFOS) plasma concentration. In total, we studied 1597 participants in the present analysis. Compared with those not included (*n* = 531), mothers included in the present analysis were more likely to be white, but other characteristics were similar (Additional file 1: Table S1). We obtained written informed consent from all participants, and the study was approved by Institutional Review Boards of participating institutions. The involvement of the Centers for Disease Control and Prevention (CDC) laboratory did not constitute engagement in human subject research.

### Selection and measurement of early pregnancy environmental exposures

We collected information on maternal cigarette smoking during pregnancy and categorized participants into three groups: 1) prenatal smokers, 2) former smokers, and 3) never smokers. At the first in-person study visit, we asked expectant mothers whether they had ever smoked, and those who smoked less than 100 cigarettes in their lifetime were classified as “never smokers.” We asked smokers whether they had smoked in the three months before learning of their pregnancy, and classified women who quit at least three months prior to pregnancy as “former smokers.” We designated those who smoked during the three months before learning of their pregnancy (i.e., likely smoked in very early pregnancy) or reported smoking on the first or second trimester questionnaire or delivery interview as “prenatal smokers.” The majority (65%) of prenatal smokers quit during the first trimester, and thus were primarily early pregnancy smokers. Therefore, in secondary analyses, we also considered the subset of prenatal smokers who continued smoking beyond the first trimester and termed this group “continued smokers.”

We estimated prenatal exposure to several types of traffic pollution in Project Viva [black carbon, PM_2.5_ (PM ≤ 2.5 μm in aerodynamic diameter), neighborhood traffic density, home roadway proximity] [3]. In the present study, we evaluated prenatal exposure to black carbon because this measure of the black particles primarily emitted by traffic has been more strongly associated with health risks such as mortality and childhood asthma, as compared to other traffic-related pollution indicators like PM [23]. In addition, in Project Viva, black carbon was more strongly associated with low fetal growth than other markers of traffic pollution [3]. We estimated daily ambient black carbon exposure at the address each participant provided at the time of enrollment (median 10 weeks gestation). We used a validated spatiotemporal regression model, which included measures of land use, GIS location, daily meteorological factors, day of week and season, and interaction terms of land use measures and meteorological factors as predictors [24, 25]. An “out-of-sample” cross-validation showed a correlation of 0.73 between predicted and monitor-observed daily black carbon concentration [25]. We analyzed first trimester black carbon [average daily exposures from date of the last menstrual period (LPM) to the 93rd day after LPM] for consistency in timing with the other early pregnancy exposures in this analysis.

We also measured maternal plasma concentrations of several PFASs [PFOS, perfluorooctanoate (PFOA), perfluorohexane sulfonate (PFHxS), perfluorononanoate (PFNA), 2-(*N*-ethyl-perfluorooctane sulfonamido) acetate, 2-(*N*-methyl-perfluorooctane sulfonamido) acetate, perfluorodecanoate, and perfluorooctane sulfonamide] in early pregnancy in Project Viva [26]. We evaluated PFOS in our primary analysis because as compared to other measured PFASs, mothers in Project Viva had the highest average concentration of PFOS plasma concentrations. Likewise, PFOS has consistently been the PFAS with the highest mean serum concentrations observed in the U.S. general population since these chemicals were in peak production and detected in the National Health and Nutrition Examination Survey’s participants in 1999–2000 [15, 27]. In an a priori secondary analysis, we examined PFNA because we have previously observed the strongest inverse association between PFNA and fetal growth as compared to other PFASs in our cohort [4]. As compared to PFOS, PFNA has a narrower range of concentrations (i.e., smaller IQR and lower mean concentration), but the coefficients of variation are relatively similar (i.e., 56.9 for PFOS and 55.1 for PFNA). Although PFOA is one of the most common studied PFAS, we found null associations between maternal PFOA plasma concentration and BW/GA in Project Viva and thus did not evaluate PFOA in the present analysis. We collected maternal plasma samples during the first prenatal visit, and samples were shipped to the CDC (Atlanta, GA) for analysis, as previously described [26]. Briefly, staff at CDC quantified PFAS concentration in maternal plasma using on-line solid-phase extraction coupled to isotope dilution high performance liquid chromatography mass spectrometry [28]. The limit of detection (LOD) was 0.2 ng/mL for PFOS and 0.1 ng/mL for PFNA; we replaced values below the LOD with the LOD/√2. Two (0.1%) women had PFOS plasma concentrations below the LOD and 22 (1.4%) women had PFNA plasma concentrations below the LOD.

### Measurement of outcome

To compute BW/GA z-score, we abstracted birth weight (in grams) and date of delivery from the hospital medical record. We obtained infant sex from delivery interview. We calculated length of gestation (in days) by subtracting the date of the LMP from the date of delivery, except in cases in which the length of gestation based on the second trimester ultrasound was more than 10 days different from that by the LMP. In these cases, we used the ultrasound result. Using these data, we calculated sex-specific BW/GA z-score based on U.S. national reference data [29].

### Measurement of covariates

We collected information on maternal age, education, race/ethnicity, parity, and household income using questionnaires at study enrollment. We calculated pre-pregnancy body mass index (BMI) from self-reported weight and height. As there is potential for the relationship between PFAS concentrations and fetal growth to be confounded by pregnancy physiology [30], we assessed creatinine in the same maternal plasma sample used for PFAS quantification to calculate glomerular filtration rate (GFR) and measured plasma albumin to assess plasma expansion, as described previously [26].

### Statistical analyses

We first fit unadjusted, followed by covariate-adjusted, linear regression models to examine the association of each prenatal exposure (self-reported maternal prenatal smoking, residential black carbon levels during the first trimester, and PFOS plasma concentration during early pregnancy) individually with BW/GA z-score. Next, we fit a covariate-adjusted additive model that included all three exposures simultaneously. For smoking status, we combined the never and former smoker categories because characteristics of these women were similar and combining the two categories did not appreciably change results. To ease interpretability and comparison, we present both first trimester black carbon and prenatal PFOS plasma concentration associations per interquartile range (IQR). We adjusted for covariates potentially associated with the three prenatal environmental exposures [3, 26, 31] and/or fetal growth [32]. These covariates included maternal age at enrollment (continuous), maternal pre-pregnancy BMI (continuous), race/ethnicity (white, black, Hispanic, Asian, or other), education (with or without a college degree), parity (nulliparous or multiparous), season of birth (continuous sine and cosine of date), and date of birth (continuous). Inclusion of household income, maternal GFR, plasma albumin, and week of gestation did not considerably change effect estimates (> 10%) and were not significant in adjusted models, and thus we did not include these covariates in final models. We did not adjust for gestational glucose tolerance status or gestational weight gain because these may be on the causal pathway between early pregnancy exposures and fetal growth and therefore may introduce collider bias [33]. Ninety-nine percent of participants (*n* = 1577) had complete covariate information for the multivariate regression models.

Next, we evaluated interactions between the three early pregnancy environmental exposures in the adjusted model. Using a sequential model building approach, we fit covariate-adjusted multi-pollutant models to examine all possible two-way interactions between pollutants (smoking*black carbon, smoking*PFOS, and black carbon*PFOS). Using a likelihood ratio test, we calculated a chi-square *p*-value to compare each two-way interaction model separately to the main effects additive model. If the likelihood ratio test was significant (*p* < 0.05), indicating that the more complex model was a better fit, we used this model as the new likelihood ratio test reference as we continued testing other models with one or multiple two-way interactions, and eventually compared it to a final model with all two-way interactions and a three-way interaction (smoking*black carbon*PFOS). We thus identified a final best-fit model and present individual and interaction effect estimates.

Although dichotomizing exposures and outcomes can improve interpretability of interaction effect estimates [34], we opted to represent the black carbon and PFAS exposures and the outcome (BW/GA z-score) as continuous variables to maximize power. Then, to improve interpretability, we used the best-fit model to predict the BW/GA z-score of an infant born to a “typical” Project Viva mother [based on mean age (31.8 years) and pre-pregnancy BMI (25.0 kg/m^2^) and modal education (college graduate), race/ethnicity (white), and parity (multiparous) woman] according to select levels of exposure to the three environmental toxicants in early pregnancy. We present extreme high and low exposure scenarios to capture the full range of model-predicted birth size in infants of exposed versus unexposed mothers, although most mothers have more moderate exposures. We considered “high” exposure to be 1) prenatal smoking, 2) first trimester black carbon exposure at the 90th percentile (1.08 μg/m^3^), and 3) prenatal PFOS plasma concentration at the 90th percentile (47.0 ng/mL). We considered “low” exposure to be 1) no prenatal smoking, 2) first trimester black carbon exposure at the 10th percentile (0.43 μg/m^3^), and 3) prenatal PFOS plasma concentration at the 10th percentile (14.0 ng/mL). We predicted infant BW/GA z-score for different mixture combinations of high versus low prenatal exposures. Next, we used birth weight percentiles for a white infant born at term from U.S. national reference data [29] to convert the predicted BW/GA z-scores to predicted birth weight.

In a secondary analysis, we re-ran the model building process but considered prenatal smokers to be the subset of women who smoked throughout pregnancy [continued smokers: *n* (%) = 71 (35%)] with all other women (including those who quit smoking during the first trimester) as the reference group. As compared to early pregnancy smoking, continued smoking throughout pregnancy has been more strongly associated with low birth size [6, 7, 35], but we considered continued smokers in the secondary rather than primary analysis due to limited power to detect an interaction based on the relatively small number of continued smokers. We also re-ran the model building process described above considering PFNA rather than PFOS because in Project Viva it was more strongly associated with smaller BW/GA.

We used the gmodels package in R 3.3.2 (Vienna, Austria) to calculate predicted BW/GA z-scores. For all other analyses, we used SAS version 9.4 (Cary, NC).

## Results

Of the 1597 mothers included in the analytic dataset, mean (standard deviation [SD]) maternal age at the time of enrollment was 31.8 (5.2) years. Sixty-five percent of mothers were college graduates, 49% nulliparous, 69% white, and 13% prenatally smoked. Mean (SD) residential black carbon level during the first trimester was 0.8 (0.3) μg/m^3^, prenatal PFOS plasma concentration was 29.1 (16.5) ng/mL, and BW/GA z-score was 0.19 (0.96) (Table 1).

**Table 1:** Participant characteristics overall (*n* = 1597) and by maternal smoking status, quartiles of first trimester residential black carbon, and quartiles of prenatal perfluorooctane sulfonate (PFOS) plasma concentration

		Prenatal Environmental Exposures									
		Prenatal smoking		First trimester residential black carbon^a^				PFOS plasma concentration^b^			
	Overall	No	Yes	Q1	Q2	Q3	Q4	Q1	Q2	Q3	Q4
	*n* = 1597	*n* = 1391	*n* = 206	*n* = 399	*n* = 399	*n* = 400	*n* = 399	*n* = 400	*n* = 396	*n* = 403	*n* = 398
	Mean (SD) or %	Mean (SD) or %		Mean (SD) or %				Mean (SD) or %			
Maternal characteristics											
Age at enrollment (years)	31.8 (5.2)	32.2 (5.0)	29.2 (5.8)	32.9 (4.3)	32.2 (5.2)	31.5 (5.4)	30.7 (5.6)	32.5 (5.1)	31.8 (5)	31.6 (5.3)	31.5 (5.3)
Pre-pregnancy BMI (kg/m^2^)	25.0 (5.6)	24.9 (5.4)	26.1 (6.2)	25.1 (5.5)	25.0 (5.1)	25.1 (5.8)	24.9 (5.7)	24.4 (5.3)	24.8 (5.4)	25.5 (5.8)	25.3 (5.6)
College graduate (%)	65	69	34	75	70	59	56	73	62	64	61
Nulliparous (%)	49	48	55	43	45	50	57	37	45	54	60
Race/ethnicity (%)											
White	69	69	65	86	73	65	51	70	71	66	67
Black	15	16	14	7	13	18	25	12	14	18	18
Hispanic	7	6	11	4	6	7	12	9	7	7	6
Asian	5	5	4	3	6	4	7	5	3	5	6
Other	4	4	6	1	4	7	5	4	5	4	3
Maternal prenatal exposures											
Prenatal smoking (%)	13	0	100	12	10	14	17	11	13	14	14
First trimester black carbon (μg/m^3^)	0.8 (0.3)	0.7 (0.3)	0.8 (0.3)	0.4 (0.1)	0.7 (0.1)	0.8 (0.0)	1.1 (0.2)	0.7 (0.2)	0.8 (0.3)	0.8 (0.3)	0.8 (0.3)
PFOS plasma concentration (ng/mL)	29.1 (16.5)	29.0 (16.9)	29.6 (14.1)	29.1 (16.8)	28.3 (15.2)	29.1 (16.9)	30.7 (5.6)	14.3 (3.6)	22.3 (1.9)	29.8 (2.7)	50.0 (19.2)
PFNA plasma concentration (ng/mL)	0.7 (0.4)	0.8 (0.4)	0.7 (0.3)	0.7 (0.4)	0.7 (0.4)	0.7 (0.5)	0.8 (0.4)	0.5 (0.3)	0.6 (0.2)	0.8 (0.3)	1.0 (0.5)
Infant characteristics											
Female (%)	48	48	45	47	48	48	47	48	48	49	46
BW/GA z-score	0.19 (0.96)	0.22 (0.94)	0.02 (1.08)	0.36 (0.92)	0.24 (0.96)	0.12 (0.97)	0.03 (0.98)	0.30 (0.98)	0.18 (0.98)	0.17 (0.95)	0.10 (0.94)
Birth weight (g)	3474 (591)	3484 (585)	3403 (626)	3560 (571)	3519 (532)	3424 (644)	3391 (597)	3562 (544)	3457 (652)	3477 (544)	3398 (609)
Gestational age (weeks)	39.4 (1.9)	39.4 (2.0)	39.5 (1.8)	39.5 (1.9)	39.5 (1.6)	39.4 (2.2)	39.4 (2.0)	39.6 (1.5)	39.4 (2.3)	39.5 (1.7)	39.2 (2.2)

*Abbreviations BMI* Body mass index, *BW/GA* birth weight-for-gestational age, *PFOS* perfluorooctane sulfonate, *PFNA* perfluorononanoate, *Q1 to 4* Quartiles 1 to 4

Missing in overall cohort: 12 pre-pregnancy BMI, 16 education & race/ethnicity

^a^First trimester black carbon quartile ranges: Q1 (0.13–0.56 μg/m^3^); Q2 (0.57–0.75 μg/m^3^); Q3 (0.76–0.92 μg/m^3^); and Q4 (0.93–1.71 μg/m^3^)

^b^PFOS plasma concentration quartile ranges: Q1 [<limit of detection (0.1)-18.8 ng/mL]; Q2 (18.9–25.6 ng/mL); Q3 (25.7–34.9 ng/mL); and Q4 (35.0–185.0 ng/mL)

As compared to non-smokers, mothers who smoked during early pregnancy were less educated, younger, had a higher pre-pregnancy BMI, and were less likely to be white. As compared to prenatal smokers, non-smokers had slightly lower exposure to black carbon (mean 0.80 μg/m^3^ for smokers versus 0.75 μg/m^3^ for non-smokers; *p* < 0.01), no difference in PFOS plasma concentrations (mean 29.6 ng/mL for smokers versus 29.0 ng/mL for non-smokers; *p* = 0.65), and no difference in PFNA plasma concentrations (mean 0.7 ng/mL for smokers versus 0.8 ng/mL for non-smokers; *p* = 0.06) (Table 1). Mean residential black carbon concentration was not correlated with PFAS plasma concentrations [Spearman’s *r* = 0.02 for PFOS (*p* = 0.35) and PFNA (*p* = 0.54)].

In covariate-adjusted single-pollutant models, higher black carbon exposure during the first trimester was associated with a 0.08 reduction in BW/GA z-score [95% confidence interval (CI): − 0.15, − 0.01] per IQR increment (Table 2). Also, higher maternal PFOS plasma concentration and prenatal smoking were associated with lower fetal growth, although confidence intervals crossed the null [BW/GA z-score of − 0.03 (95% CI: -0.07, 0.02) per IQR increment in PFOS and − 0.09 (95% CI: -0.24, 0.05) for infants of smokers versus non-smokers] (Table 2).

**Table 2:** Covariate-adjusted associations^a^ of prenatal smoking, first trimester residential black carbon, and prenatal perfluorooctane sulfonate (PFOS) plasma concentration and their interactions with birth weight-for-gestational age (BW/GA) z-score

		Smoking*BC	Smoking*PFOS	BC*PFOS	LRT χ^2^ *p*-value^b^
	Change in BW/GA z-score (95% CI)	Interaction β (95% CI)			
Single-pollutant exposure models					
Prenatal smoking (Y/N)	−0.09 (−0.24, 0.05)				
First trimester black carbon (per IQR)^b^	− 0.08 (− 0.15, − 0.01)				
Prenatal PFOS plasma concentration (per IQR)^b^	− 0.03 (− 0.07, 0.02)				
Multi-pollutant exposure models					
Main effects (Additive model)	Smoking: −0.09 (− 0.23, 0.06)				Reference
	BC: −0.08 (− 0.15, − 0.01)				
	PFOS: −0.03 (− 0.08, 0.02)				
Main effects + Smoking*BC	Smoking: 0.04 (−0.39, 0.47)	−0.06 (− 0.24, 0.13)			0.53
	BC: −0.07 (− 0.15, 0.00)				
	PFOS: −0.03 (− 0.08, 0.02)				
Main effects + Smoking*PFOS	Smoking: −0.25 (− 0.57, 0.06)		0.09 (− 0.06, 0.24)		0.24
	BC: −0.08 (− 0.15, − 0.01)				
	PFOS: −0.04 (− 0.09, 0.01)				
Main effects + BC*PFOS	Smoking: −0.08 (− 0.22, 0.06)			0.05 (−0.00, 0.11)	0.07 ^c^
	BC: −0.18 (− 0.31, − 0.05)				
	PFOS: − 0.14 (− 0.27, − 0.01)				

*Abbreviations* χ^2^ chi-square, *BC* black carbon, *BW/GA* birth weight-for-gestational age, *CI* confidence interval, *IQR* interquartile range, *LRT* likelihood ratio test, *PFOS* perfluorooctane sulfonate

Estimates with 95% confidence intervals that do not cross the null are bolded. Likelihood ratio test (LRT) chi-square (χ^2^) *p*-value < 0.05 indicates that the more complex model is a better fit than the reference model

^a^Adjusted for maternal age, race/ethnicity, education, pre-pregnancy body mass index, and parity. The first trimester black carbon single-pollutant exposure model was additionally adjusted for season and date of birth, and these covariates were included in all multi-pollutant exposure models

^b^Interquartile range increments: 0.36 μg/m^3^ for first trimester black carbon and 16.1 ng/mL for prenatal PFOS plasma concentration

^c^When we subsequently compared models with multiple two-way interactions and the three-way interaction to the best-fit model (main effects additive model), all *p*-values were > 0.05

In the covariate-adjusted multi-pollutant main effects additive model, effect sizes and significance for all three exposures in relation to fetal growth were similar to those in the single-pollutant models (Table 2).

None of the two-way interaction terms were significantly associated with fetal growth, and the likelihood ratio test confirmed that the multi-pollutant additive model without any interaction terms was the best-fit model (Table 2). Additionally, the three-way interaction was not statistically significant [0.16 (95% CI: -0.03, 0.35)] (data not shown).

**Fig. 1:**
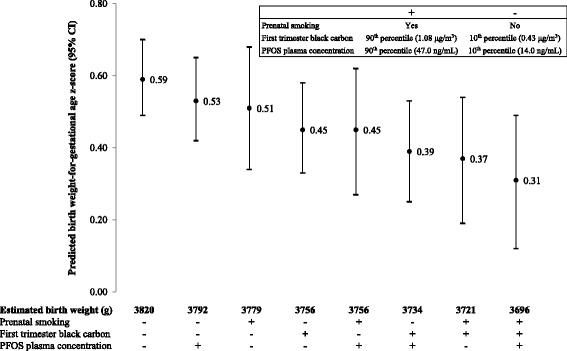
Predicted birth weight-for-gestational age z-scores for different scenarios of prenatal smoking, first trimester residential black carbon, and prenatal perfluorooctane sulfonate (PFOS) plasma concentration. Error bars represent 95% confidence intervals. Estimates are based on a model adjusted for maternal age, race/ethnicity, education, pre-pregnancy body mass index, parity, season of birth, and date of birth; are for a “typical” (average) Project Viva participant: 31.8 years old, a college graduate, white, multiparous, and had a pre-pregnancy BMI of 25.0 kg/m^2^; and are for a white full-term (40 weeks) offspring [29].

To aid in interpretability, we used the best-fit model to calculate predicted BW/GA z-scores for extreme exposure scenarios. As shown in Fig. 1, infants of mothers with low levels of all three exposures (10th percentile of black carbon and PFOS, and non-smokers) had a predicted BW/GA z-score of 0.59 (95% CI: 0.49, 0.70) versus 0.31 (95% CI: 0.12, 0.49) for infants of mothers with high levels of all exposures (90th percentile of black carbon and PFOS, and smokers), corresponding to an estimated birth weight difference of 124 g for a white infant born at term [29]. Infants with mixtures of some high and some low exposures had BW/GA z-scores between 0.31 and 0.59 (Fig. 1).

In secondary analyses, infants of mothers who smoked throughout pregnancy (continued smokers) had lower BW/GA z-scores [− 0.40 (95% CI: -0.63, − 0.18)] compared to infants of mothers who were non-smokers, former smokers, or early pregnancy smokers, in the best-fit main effects additive model (data not shown).

Maternal plasma concentration of PFNA was more strongly associated with lower BW/GA z-score as compared to PFOS [− 0.05 (95% CI: -0.10, − 0.01) per IQR increment in PFNA versus − 0.03 (95% CI: -0.07, 0.02) per IQR increment in PFOS in the covariate-adjusted single-pollutant model]. The best-fit, multi-pollutant model included positive two-way interactions between PFNA plasma concentration and both prenatal smoking and black carbon exposure (Additional file 1: Table S2). Thus, the BW/GA z-score for infants of mothers with high PFNA concentration and exposed to high black carbon or with high PFNA concentration and exposed to maternal smoking, was not as low as expected based on the individual associations of each of the pollutants with fetal growth. For example, in the extreme exposure scenarios, infants of non-smoking mothers with low black carbon and high PFNA concentration had a predicted BW/GA z-score of 0.41 (95% CI: 0.27, 0.55) and infants of non-smoking mothers with high black carbon and low PFNA concentration had a predicted BW/GA z-score of 0.43 (95% CI: 0.29, 0.58), but infants of non-smoking mothers with both high black carbon and high PFNA concentration had a predicted BW/GA z-score of 0.39 (95% CI: 0.25, 0.53) (Additional file 1: Figure S1).

## Discussion

In our analysis of a large, prospective cohort of mother-infant pairs, we support previously reported findings of lower birth size with higher prenatal maternal exposure to smoking, black carbon, and PFOS in early pregnancy. We additionally found that simultaneous exposure to these compounds had an additive rather than interactive effect on fetal growth. However, infants prenatally exposed to PFNA and either maternal smoking or black carbon had a higher birth size than expected based on the individual associations with fetal growth.

Associations between higher prenatal exposures to maternal smoking, air pollution, and PFASs with lower birth size have been previously reported in the Project Viva cohort [3–5] and others [6–10]. In the analytic cohort of the present study, while all three exposures were associated with lower BW/GA, confidence intervals crossed the null for early pregnancy smoking and PFOS exposure. Other cohorts have shown that maternal smoking in mid to late but not early pregnancy leads to decrements in fetal growth [6, 7, 35], on the order of 175–200 g lower birth weight [6]. Consistent with this, in the Project Viva cohort, prenatal smoking was most strongly associated with low fetal growth for women who smoked throughout pregnancy (versus those who did not), corresponding to a decrement of 170 g in birth weight for a full term white infant.

An association between higher prenatal air pollution and lower birth weight has been shown in numerous previous studies (e.g., [36–38]), with a pooled-effect of 23.4 g (95% CI: -45.5, − 1.4) lower birth weight per 10 μg/m^3^ of ambient PM_2.5_ (of which black carbon is a component) in a recent meta-analysis [8]. For comparison, in the present analysis, we estimated a 36 g decrement in birth weight (for a white term infant at the 50th percentile of BW/GA z-score) for each IQR increment in first trimester black carbon exposure.

PFASs may reduce fetal growth via activation of PPAR or through structural similarity to free fatty acids which may modulate lipid and glucose homeostasis [20], impair placental transfer of lipids and glucose, and thereby lead to oxidative stress [39]. A meta-analysis of nine studies found a 19 g (95% CI: -30, − 8) lower birth weight for each ng/mL increment in cord serum or maternal plasma PFOA concentration [10]. This translates to a 49 g lower birth weight for each IQR increment of PFOA using the IQR from U.S. females in NHANES during 1999–2000 [40]. The present study found more modest associations with PFOS and PFNA. For a white term infant in the 50th percentile of BW/GA z-score, we estimated a 10 g decrement in birth weight for each IQR increment in maternal PFOS plasma concentration, although confidence intervals included the null. We estimated a larger, 19 g decrement in birth weight for each IQR increment in maternal PFNA plasma concentration.

The present study adds to the existing literature by examining the extent to which early pregnancy exposure to maternal smoking, black carbon, and select PFASs interact to alter fetal growth. We found that while PFOS did not modify the relationships of maternal smoking or black carbon with fetal growth, PFNA attenuated the associations of smoking and black carbon on lower BW/GA. This finding raises the possibility that PFNA may act antagonistically on similar biological pathways as maternal smoking and black carbon. For example, placental inflammation as a result of air pollution exposure during pregnancy may lead to impaired nutrient exchange to the fetus and lower fetal growth [12]. PFAS may counter pollution-induced placental inflammation by inhibiting inflammatory gene expression via activation of PPAR [18, 19], thus leading to a higher birth weight than otherwise expected from the concurrent exposures. Consistent with our finding of an interaction with PFNA but not PFOS, in mouse and human models, PFNA activated PPAR-alpha at lower doses and with higher potency than shorter chain carboxylates, such as PFOA, or sulfonates, like PFOS [41]. Nevertheless, our finding requires replication in other cohorts.

We found that although maternal smoking and black carbon independently lowered fetal growth, they did not act synergistically. The prior literature on the association between traffic pollution and birth outcomes for woman prenatally exposed versus not exposed to cigarette smoke is conflicting and limited. Specifically, in a study of over 200,000 Canadian births, maternal smoking attenuated the association between air pollution exposure and low birth weight [42], whereas a study of over 600,000 U.S. births found the association between high prenatal traffic-related carbon monoxide exposure and low birth weight to be larger among smoking versus non-smoking mothers [43]. In a U.S. urban cohort of over 200 non-smoking pregnant women, passive smoke exposure and a blood marker of ambient air pollution (benzo[a]pyrene-DNA adducts) synergistically lowered birth weight, although there were not independent associations between either exposure with birth weight [44]. Even when we considered maternal smoking throughout pregnancy, which was more strongly associated with low fetal growth than early pregnancy smoking in the Project Viva cohort, we observed no interaction with black carbon on fetal growth. Although cigarette smoking impairs maternal immune function [45] and induces fetal hypoxia [13], which could potentially cause a fetus to be more vulnerable to the effects of other pollutants like black carbon, mothers in our cohort may not have been heavy enough smokers to significantly weaken responses to these other toxicants. Alternatively, the fact that cigarette smoke and black carbon exposure impact fetal growth through similar mechanisms of action may explain their additive rather than synergistic or antagonistic mechanism of action. Furthermore, we may not have had the power to detect an interaction between maternal smoking and black carbon exposure on fetal growth because only a small proportion of the cohort smoked during pregnancy and variability of ambient black carbon concentrations in the Boston area is relatively low. Power to detect an interaction between these two exposures may have been further limited because of exposure misclassification. Social desirability bias may have introduced some misclassification into our questionnaire measure of maternal smoking, and we did not have data on work location or time at home which could have improved the accuracy of black carbon exposure estimates [46]. In contrast, PFAS exposure was less likely to be misclassified, as these compounds have long half-lives, and we measured concentrations in plasma.

Although our current analysis uniquely studied how maternal smoking, black carbon, and PFASs may interact to influence birth size, prior studies have focused on how mixtures of other pollutants may impact birth weight. One study of about 1000 European mother-infant pairs observed a stronger negative cross-sectional association between cord blood dioxin-like activity and birth weight among infants of women who smoked during pregnancy versus non-smokers, but the interaction was not significant [47]. Another study of over 1000 mother-infant pairs from Greenland, Poland, and Ukraine observed greater birth weight reductions for infants of smokers concomitantly prenatally exposed to PFOA and di(2-ethylhexyl) phthalate (DEHP) versus non-smokers exposed to these two chemicals [48]. Additionally, a study of about 250 Flemish mother-infant pairs found a larger decrement in birth weight in infants simultaneously exposed to arsenic, PFOA, lead, cadmium, and DEHP as compared to individual exposures, although they did not test for interactions between compounds [49].

The multi-pollutant model approach allows for evaluation of joint effects and is generally expected to minimize co-exposure confounding [50]. However, as is the case with single-pollutant models, misspecification due to adjustment for irrelevant or collider variables, or lack of adjustment for confounder variables could lead to bias [51, 52]. Although collinearity between exposures in a multi-pollutant model could decrease precision of effect estimates [50], this was not a limitation of the present analysis, as the exposures we studied were not tightly correlated.

Generalizability is a limitation of our cohort, as participants are primarily of moderately high socioeconomic status and all resided in Massachusetts at enrollment. In addition, although we limited our primary analysis to early pregnancy exposures for which we have the most robust data, timing of exposure could alter independent and combined effects of these pollutants on fetal growth. Also, to target our analysis and limit multiple comparisons, we focused on one air pollutant (black carbon) and one PFAS (PFOS) in our primary analyses, although pregnant women are exposed to several types of air pollutants and PFAS concurrently. Our observation that PFNA had stronger independent and combined effects on fetal growth than PFOS suggests that specific PFASs may act differently. However, PFNA plasma concentrations have a relatively narrow range in our cohort, and thus we cannot generalize our findings to women with higher PFNA concentrations.

Strengths of our study include use of a large prospective cohort with measurements of multiple prenatal environmental exposures known to influence fetal growth. Additionally, most environmental epidemiological studies examine the effect of a single pollutant on health outcomes, despite the fact that individuals are exposed to multiple pollutants concurrently [21]. We present an approach, of examining multi-pollutant models with two-way and three-way interactions, to investigate health effects related to joint exposure to multiple toxicants.

## Conclusion

We observed that concurrent early pregnancy exposures to maternal smoking, residential black carbon, and PFOS were associated with additive reductions in fetal growth, while exposure to PFNA attenuated the associations of smoking and black carbon exposure on low birth size, in a Boston-area cohort. Because a developing fetus is exposed to mixtures of chemicals, it is critical to continue to evaluate the role of multiple pollutant exposures in future studies. Further investigation of the effect of mixtures of in utero exposures on health outcomes may provide insight into potential biological pathways of action.

## Additional file

Additional file 1: Supplemental Material. (DOCX 37 kb)

## Abbreviations

BMI: Body mass index
BW/GA: Birth weight-for-gestational age
CDC: Centers for Disease Control and Prevention
CI: Confidence interval
DEHP: di(2-ethylhexyl)
GFR: Glomerular filtration rate
IQR: Interquartile range
LMP: Last menstrual period
LOD: Limit of detection
PFAS: Perfluoroalkyl substances
PFHxS: Perfluorohexane sulfonate
PFNA: Perfluorononanoate
PFOA: Perfluorooctanoate
PFOS: Perfluorooctane sulfonate
PM: Particulate matter
PPAR: Peroxisome proliferator-activated receptor
SD: Standard deviation

## References

[1] Nardozza LM, Caetano AC, Zamarian AC, Mazzola JB, Silva CP, Marcal VM (2017). Fetal growth restriction: current knowledge. Arch Gynecol Obstet.

[2] Barker DJ (2006). Adult consequences of fetal growth restriction. Clin Obstet Gynecol.

[3] Fleisch AF, Rifas-Shiman SL, Koutrakis P, Schwartz JD, Kloog I, Melly S (2015). Prenatal exposure to traffic pollution: associations with reduced fetal growth and rapid infant weight gain. Epidemiology.

[4] Sagiv SK, Rifas-Shiman SL, Fleisch AF, Webster TF, Calafat AM, Ye X, et al. Early pregnancy Perfluoroalkyl substance plasma concentrations and birth outcomes in project viva: confounded by pregnancy hemodynamics? Am J Epidemiol. 2017. 10.1093/aje/kwx332. [Epub ahead of print]. 10.1093/aje/kwx332 PMC5884712 29155920

[5] Fleisch AF, Rifas-Shiman SL, Rokoff LB, Hivert MF, Mantzoros CS, Oken E (2017). Associations of maternal prenatal smoking with umbilical cord blood hormones: the project viva cohort. Metabolism.

[6] Lumley J (1987). Stopping smoking. Br J Obstet Gynaecol.

[7] Reeves S, Bernstein I (2008). Effects of maternal tobacco-smoke exposure on fetal growth and neonatal size. Expert Rev Obstet Gynecol.

[8] Stieb DM, Chen L, Eshoul M, Judek S (2012). Ambient air pollution, birth weight and preterm birth: a systematic review and meta-analysis. Environ Res.

[9] Bach CC, Bech BH, Brix N, Nohr EA, Bonde JP, Henriksen TB (2015). Perfluoroalkyl and polyfluoroalkyl substances and human fetal growth: a systematic review. Crit Rev Toxicol.

[10] Johnson PI, Sutton P, Atchley DS, Koustas E, Lam J, Sen S (2014). The navigation guide - evidence-based medicine meets environmental health: systematic review of human evidence for PFOA effects on fetal growth. Environ Health Perspect.

[11] Invernizzi G, Ruprecht A, Mazza R, Rossetti E, Sasco A, Nardini S (2004). Particulate matter from tobacco versus diesel car exhaust: an educational perspective. Tob Control.

[12] Kannan S, Misra DP, Dvonch JT, Krishnakumar A (2006). Exposures to airborne particulate matter and adverse perinatal outcomes: a biologically plausible mechanistic framework for exploring potential effect modification by nutrition. Environ Health Perspect.

[13] Rogers JM (2008). Tobacco and pregnancy: overview of exposures and effects. Birth Defects Res C Embryo Today.

[14] Lindstrom AB, Strynar MJ, Libelo EL (2011). Polyfluorinated compounds: past, present, and future. Environ Sci Technol.

[15] Centers for Disease Control and Prevention: Fourth National Report on Human Exposure to Environmental Chemicals. In: Updated Tables, January 2017, Volume One. Atlanta, GA. 2017.

[16] Lau C, Anitole K, Hodes C, Lai D, Pfahles-Hutchens A, Seed J (2007). Perfluoroalkyl acids: a review of monitoring and toxicological findings. Toxicol Sci.

[17] Vanden Heuvel JP, Thompson JT, Frame SR, Gillies PJ (2006). Differential activation of nuclear receptors by perfluorinated fatty acid analogs and natural fatty acids: a comparison of human, mouse, and rat peroxisome proliferator-activated receptor-alpha, −beta, and -gamma, liver X receptor-beta, and retinoid X receptor-alpha. Toxicol Sci.

[18] Stienstra R, Duval C, Muller M, Kersten S (2007). PPARs, obesity, and inflammation. PPAR Res.

[19] Abbott BD (2009). Review of the expression of peroxisome proliferator-activated receptors alpha (PPAR alpha), beta (PPAR beta), and gamma (PPAR gamma) in rodent and human development. Reprod Toxicol.

[20] DeWitt JC, Shnyra A, Badr MZ, Loveless SE, Hoban D, Frame SR (2009). Immunotoxicity of perfluorooctanoic acid and perfluorooctane sulfonate and the role of peroxisome proliferator-activated receptor alpha. Crit Rev Toxicol.

[21] Woodruff TJ, Zota AR, Schwartz JM (2011). Environmental chemicals in pregnant women in the United States: NHANES 2003-2004. Environ Health Perspect.

[22] Oken E, Baccarelli AA, Gold DR, Kleinman KP, Litonjua AA, De Meo D (2015). Cohort profile: project viva. Int J Epidemiol.

[23] Janssen NA, Hoek G, Simic-Lawson M, Fischer P, van Bree L, ten Brink H (2011). Black carbon as an additional indicator of the adverse health effects of airborne particles compared with PM10 and PM2.5. Environ Health Perspect.

[24] Gryparis A, Coull BA, Schwartz J, Suh HH (2007). Semiparametric latent variable regression models for spatiotemporal modelling of mobile source particles in the greater Boston area. J R Stat Soc: Ser C: Appl Stat.

[25] Zanobetti A, Coull BA, Gryparis A, Kloog I, Sparrow D, Vokonas PS (2014). Associations between arrhythmia episodes and temporally and spatially resolved black carbon and particulate matter in elderly patients. Occup Environ Med.

[26] Sagiv SK, Rifas-Shiman SL, Webster TF, Mora AM, Harris MH, Calafat AM (2015). Sociodemographic and perinatal predictors of early pregnancy per- and Polyfluoroalkyl substance (PFAS) concentrations. Environ Sci Technol.

[27] Kato K, Wong LY, Jia LT, Kuklenyik Z, Calafat AM (2011). Trends in exposure to polyfluoroalkyl chemicals in the U.S. population: 1999-2008. Environ Sci Technol.

[28] Kato K, Basden BJ, Needham LL, Calafat AM (2011). Improved selectivity for the analysis of maternal serum and cord serum for polyfluoroalkyl chemicals. J Chromatogr A.

[29] Oken E, Kleinman KP, Rich-Edwards J, Gillman MW (2003). A nearly continuous measure of birth weight for gestational age using a United States national reference. BMC Pediatr.

[30] Savitz DA (2007). Guest editorial: biomarkers of perfluorinated chemicals and birth weight. Environ Health Perspect.

[31] Shoff C, Yang TC (2013). Understanding maternal smoking during pregnancy: does residential context matter?. Soc Sci Med.

[32] Kramer MS (1987). Determinants of low birth weight: methodological assessment and meta-analysis. Bull World Health Organ.

[33] Cole SR, Platt RW, Schisterman EF, Chu H, Westreich D, Richardson D (2010). Illustrating bias due to conditioning on a collider. Int J Epidemiol.

[34] Knol MJ, VanderWeele TJ (2012). Recommendations for presenting analyses of effect modification and interaction. Int J Epidemiol.

[35] Lindley AA, Becker S, Gray RH, Herman AA (2000). Effect of continuing or stopping smoking during pregnancy on infant birth weight, crown-heel length, head circumference, ponderal index, and brain:body weight ratio. Am J Epidemiol.

[36] Parker JD, Woodruff TJ, Basu R, Schoendorf KC (2005). Air pollution and birth weight among term infants in California. Pediatrics.

[37] Parker JD, Woodruff TJ (2008). Influences of study design and location on the relationship between particulate matter air pollution and birthweight. Paediatr Perinat Epidemiol.

[38] Morello-Frosch R, Jesdale BM, Sadd JL, Pastor M (2010). Ambient air pollution exposure and full-term birth weight in California. Environ Health.

[39] Herrera E, Ortega-Senovilla H (2014). Lipid metabolism during pregnancy and its implications for fetal growth. Curr Pharm Biotechnol.

[40] Calafat AM, Kuklenyik Z, Reidy JA, Caudill SP, Tully JS, Needham LL (2007). Serum concentrations of 11 polyfluoroalkyl compounds in the U.S. population: data from the National Health and nutrition examination survey (NHANES). Environ Sci Technol.

[41] Wolf CJ, Takacs ML, Schmid JE, Lau C, Abbott BD (2008). Activation of mouse and human peroxisome proliferator-activated receptor alpha by perfluoroalkyl acids of different functional groups and chain lengths. Toxicol Sci.

[42] Erickson AC, Ostry A, Chan HM, Arbour L (2016). Air pollution, neighbourhood and maternal-level factors modify the effect of smoking on birth weight: a multilevel analysis in British Columbia, Canada. BMC Public Health.

[43] Currie J, Neidell M, Schmieder JF (2009). Air pollution and infant health: lessons from New Jersey. J Health Econ.

[44] Perera FP, Rauh V, Whyatt RM, Tsai WY, Bernert JT, Tu YH (2004). Molecular evidence of an interaction between prenatal environmental exposures and birth outcomes in a multiethnic population. Environ Health Perspect.

[45] Sopori M (2002). Effects of cigarette smoke on the immune system. Nat Rev Immunol.

[46] Nethery E, Brauer M, Janssen P (2009). Time-activity patterns of pregnant women and changes during the course of pregnancy. J Expo Sci Environ Epidemiol.

[47] Vafeiadi M, Agramunt S, Pedersen M, Besselink H, Chatzi L, Fthenou E (2014). In utero exposure to compounds with dioxin-like activity and birth outcomes. Epidemiology.

[48] Lenters V, Portengen L, Rignell-Hydbom A, Jonsson BA, Lindh CH, Piersma AH (2016). Prenatal phthalate, Perfluoroalkyl acid, and organochlorine exposures and term birth weight in three birth cohorts: multi-pollutant models based on elastic net regression. Environ Health Perspect.

[49] Govarts E, Remy S, Bruckers L, Den Hond E, Sioen I, Nelen V (2016). Combined effects of prenatal exposures to environmental chemicals on birth weight. Int J Environ Res Public Health.

[50] Dominici F, Peng RD, Barr CD, Bell ML (2010). Protecting human health from air pollution: shifting from a single-pollutant to a multipollutant approach. Epidemiology.

[51] Schisterman EF, Perkins NJ, Mumford SL, Ahrens KA, Mitchell EM (2017). Collinearity and causal diagrams: a lesson on the importance of model specification. Epidemiology.

[52] Greenland S (2003). Quantifying biases in causal models: classical confounding vs collider-stratification bias. Epidemiology.

