# Supplementary material for: Cumulative exposure to environmental pollutants during early pregnancy and reduced fetal growth: the Project Viva cohort
Source: Environ Health. 2018 Feb 20;17:19. doi: 10.1186/s12940-018-0363-4 (PMC5819079; doi:10.1186/s12940-018-0363-4)
Supplement: Supplementary file 1 — Supplemental Material. (DOCX 37 kb) [file 12940_2018_363_MOESM1_ESM.docx]

**Table S1.** Characteristics of Project Viva participants in the full cohort and in the subset not included versus included in analyses

| **3^rd^ trimester BC (µg/m^3^), Mean (SD)** | **Full cohort** | | **Subset included in analyses** | | **Subset not included in analyses** | |
| --- | --- | --- | --- | --- | --- | --- |
| **3^rd^ trimester BC (µg/m^3^), Mean (SD)** | **(n=2,128)** | | **(n=1,597)**  0.69 (0.23) | | **(n=531)** | |
|  | **N** | **Mean (SD) or %** | **N** | **Mean (SD) or %** | **N** | **Mean (SD) or %** |
| **Maternal characteristics** |  |  |  |  |  |  |
| Age at enrollment (years) | 2,128 | 31.8 (5.2) | 1,597 | 31.8 (5.2) | 531 | 31.7 (5.3) |
| Pre-pregnancy BMI (kg/m^2^) | 2,112 | 24.9 (5.5) | 1,585 | 25.0 (5.6) | 527 | 24.5 (5.4) |
| College graduate (%) | 2,104 | 65 | 1,581 | 65 | 523 | 64 |
| Nulliparous (%) | 2,128 | 48 | 1,597 | 49 | 531 | 45 |
| Race/ethnicity (%) | 2,104 |  | 1,581 |  | 523 |  |
| White |  | 66 |  | 69 |  | 60 |
| Black |  | 17 |  | 15 |  | 20 |
| Hispanic |  | 7 |  | 7 |  | 8 |
| Asian |  | 6 |  | 5 |  | 8 |
| Other |  | 4 |  | 4 |  | 4 |
|  |  |  |  |  |  |  |
| **Maternal prenatal exposures** |  |  |  |  |  |  |
| Prenatal smoking (%) | 2,107 | 13 | 1,597 | 13 | 510 | 12 |
| First trimester black carbon (μg/m^3^) | 2,091 | 0.8 (0.3) | 1,597 | 0.8 (0.3) | 494 | 0.7 (0.3) |
| PFOS plasma concentration (ng/mL) | 1,645 | 29.1 (16.5) | 1,597 | 29.1 (16.5) | 48 | 28.4 (14.9) |
| PFNA plasma concentration (ng/mL) | 1,645 | 0.7 (0.4) | 1,597 | 0.7 (0.4) | 48 | 0.7 (0.4) |
|  |  |  |  |  |  |  |
| **Infant characteristics** |  |  |  |  |  |  |
| Female (%) | 2,128 | 48 | 1,597 | 48 | 531 | 51 |
| BW/GA z-score | 2,127 | 0.17 (0.97) | 1,597 | 0.19 (0.96) | 530 | 0.12 (0.99) |
| Birth weight (g) | 2,127 | 3461 (592) | 1,597 | 3474 (591) | 530 | 3423 (596) |
| Gestational age (weeks) | 2,128 | 39.4 (2.0) | 1,597 | 39.4 (1.9) | 531 | 39.4 (2.0) |
| Abbreviations: BMI – Body mass index; BW/GA – birth weight-for-gestational age; PFOS – perfluorooctane sulfonate; perfluorononanoate | | | | | | |

**Table S2.** Covariate-adjusted associations ^a^ of prenatal smoking (Y/N), first trimester residential black carbon (BC), and prenatal perfluorononanoate (PFNA) plasma concentration and their interactions with birth weight-for-gestational age (BW/GA) z-score.

|  |  | **Smoking*BC** | **Smoking*PFNA** | **BC*PFNA** | **LRT χ^2^ p-value** |
| --- | --- | --- | --- | --- | --- |
| **Single-pollutant exposure models** | **Change in BW/GA z-score (95% CI)** | **Interaction β (95% CI)** | | |  |
| Prenatal smoking | -0.09 (-0.24, 0.05) |  |  |  |  |
| First trimester black carbon (per IQR) ^c^ | **-0.08 (-0.15, -0.01)** |  |  |  |  |
| Prenatal PFNA plasma concentration (per IQR) ^b^ | **-0.05 (-0.10, -0.01)** |  |  |  |  |
| **Multi-pollutant exposure models** |  |  |  |  |  |
| Main effects additive model | Smoking: -0.09 (-0.23, 0.06) |  |  |  |  |
|  | **BC: -0.08 (-0.15, -0.01)** |  |  |  | Reference |
|  | **PFNA: -0.05 (-0.10, -0.01)** |  |  |  |  |
| Main effects + Smoking*BC | Smoking: 0.05 (-0.38, 0.47) | -0.06 (-0.25, 0.12) |  |  |  |
|  | BC: -0.07 (-0.14, 0.01) |  |  |  | 0.51 |
|  | **PFNA: -0.05 (-0.10, -0.01)** |  |  |  |  |
| Main effects + Smoking*PFNA | **Smoking: -0.39 (-0.70, -0.07)** |  | **0.17 (0.01, 0.33)** |  |  |
|  | **BC: -0.08 (-0.15, -0.00)** |  |  |  | **0.03^c^** |
|  | **PFNA: -0.07 (-0.12, -0.02)** |  |  |  |  |
| Main effects + BC*PFNA | Smoking: -0.08 (-0.23, 0.06) |  |  | **0.06 (0.00, 0.12)** |  |
|  | **BC: -0.20 (-0.34, -0.07)** |  |  |  | **0.04** |
|  | **PFNA: -0.20 (-0.34, -0.05)** |  |  |  |  |
| Main effects + Smoking*BC + Smoking*PFNA | Smoking: -0.26 (-0.77, 0.26) | -0.06 (-0.24, 0.12) | **0.17 (0.01, 0.33)** |  |  |
|  | BC: -0.07 (-0.14, 0.01) |  |  |  | 0.52 |
|  | **PFNA: -0.07 (-0.12, -0.02)** |  |  |  |  |
| Main effects + Smoking*BC + BC*PFNA | Smoking: 0.03 (-0.40, 0.46) | -0.05 (-0.23, 0.14) |  | **0.06 (0.00, 0.12)** |  |
|  | **BC: -0.19 (-0.33, -0.05)** |  |  |  | 0.63 |
|  | **PFNA: -0.19 (-0.33, -0.05)** |  |  |  |  |
| Main effects + Smoking*PFNA + BC*PFNA | **Smoking: -0.39 (-0.71, -0.08)** |  | **0.18 (0.02, 0.34)** | **0.06 (0.01, 0.12)** |  |
|  | **BC: -0.20 (-0.34, -0.07)** |  |  |  | **0.03^d^** |
|  | **PFNA: -0.21 (-0.35, -0.07)** |  |  |  |  |

Estimates with 95% confidence intervals that do not cross the null are bolded. Likelihood ratio test (LRT) chi-square (χ^2^) p-value < 0.05 indicates that the more complex model is a better fit than the reference model.

Abbreviation: χ^2^ – chi-square; BC – black carbon, BW/GA – birth weight-for-gestational age; CI – confidence interval, IQR – interquartile range; LRT – likelihood ratio test; PFNA – perfluorononanoate

^a^ Adjusted for maternal age, race/ethnicity, education, pre-pregnancy body mass index, parity. The first trimester black carbon single-pollutant exposure model was additionally adjusted for season of birth and date of birth, and these covariates were included in all of the multi-pollutant exposure models.

^b^ Interquartile range increments: 0.36 μg/m^3^ for first trimester black carbon and 0.4 ng/mL for prenatal PFNA plasma concentration

^c^ Models with two 2-way interactions were compared to the new best-fit model as a reference (main effects +Smoking*PFNA)

^d^ When we subsequently compared models with all two-way interactions and the three-way interaction to the best-fit model (main effects + Smoking*PFNA + BC*PFNA), all p-values were > 0.05.

**Figure S1.** Predicted birth weight-for-gestational age z-score for different high/low scenarios of the three prenatal environmental exposures [prenatal smoking (Y/N), first trimester residential black carbon, and prenatal perfluorononanoate (PFNA) plasma concentration] in a “typical” ^a^ Viva participant

|  | **+** | **-** |
| --- | --- | --- |
| Prenatal smoking | Yes | No |
| First trimester black carbon | 90^th^ percentile (1.08 μg/m^3^) | 10^th^ percentile (0.43 μg/m^3^) |
| PFNA plasma concentration | 90^th^ percentile (1.2 ng/mL) | 10^th^ percentile (0.3 ng/mL) |

| **Estimated birth weight (g)^b^** | **3876** | **3745** | **3756** | **3756** | **3806** | **3734** | **3638** | **3792** |
| --- | --- | --- | --- | --- | --- | --- | --- | --- |
| Prenatal smoking | **-** | **-** | **+** | **-** | **+** | **-** | **+** | **+** |
| First trimester black carbon | **-** | **-** | **-** | **+** | **-** | **+** | **+** | **+** |
| PFNA plasma concentration | **-** | **+** | **-** | **-** | **+** | **+** | **-** | **+** |
|  |  |  |  |  |  |  |  |  |

Error bars represent 95% confidence intervals

Abbreviation: CI – confidence interval; PFNA – perfluorononanoate

Model adjusted for maternal age, race/ethnicity, education, pre-pregnancy body mass index, parity, season of birth, date of birth, and the Smoking*PFNA and BC*PFNA interaction terms

^a^ “Typical” participant is 31.8 years old, a college graduate, white, multiparous and had a pre-pregnancy BMI of 25.0 kg/m^2^

^b^ For a white full-term (40 weeks) offspring (Oken et al. 2003).
